# Evaluation of the Diagnostic Accuracy of Prototype Rapid Tests for Human African Trypanosomiasis

**DOI:** 10.1371/journal.pntd.0003373

**Published:** 2014-12-18

**Authors:** Jeremy M. Sternberg, Marek Gierliński, Sylvain Biéler, Michael A. J. Ferguson, Joseph M. Ndung'u

**Affiliations:** 1 Institute of Biological and Environmental Sciences, University of Aberdeen, Aberdeen, United Kingdom; 2 College of Life Sciences, University of Dundee, Dundee, United Kingdom; 3 Foundation for Innovative New Diagnostics (FIND) Campus Biotech, Geneva, Switzerland; New York University School of Medicine, United States of America

## Abstract

**Background:**

Diagnosis of human African trypanosomiasis (HAT) remains a challenge both for active screening, which is critical in control of the disease, and in the point-of-care scenario where early and accurate diagnosis is essential. Recently, the first field deployment of a lateral flow rapid diagnostic test (RDT) for HAT, “SD BIOLINE HAT” has taken place. In this study, we evaluated the performance of “SD BIOLINE HAT” and two new prototype RDTs.

**Methodology/Principal Findings:**

The performance of “SD BIOLINE HAT” and 2 prototype RDTs was tested using archived plasma from 250 *Trypanosoma brucei gambiense* patients, and 250 endemic controls. As well as comparison of the sensitivity and specificity of each device, the performance of individual antigens was assessed and the hypothetical performance of novel antigen combinations extrapolated. Neither of the prototype devices were inferior in sensitivity or specificity to “SD BIOLINE HAT” (sensitivity 0.82±0.01, specificity 0.97±0.01, 95% CI) at the 5% margins, while one of the devices (BBI) had significantly superior sensitivity (0.88±0.03). Analysis of the performance of individual antigens was used to model new antigen combinations to be explored in development of the next generation of HAT RDTs. The modelling showed that an RDT using two recombinant antigens (rLiTat1.5 and rISG65) would give a performance similar to the best devices in this study, and would also offer the most robust performance under deteriorating field conditions.

**Conclusions/Significance:**

Both “SD BIOLINE HAT” and the prototype devices performed comparably well to one another and also to the published performance range of the card agglutination test for trypanosomiasis in sensitivity and specificity. The performance of individual antigens enabled us to predict that an all-recombinant antigen RDT can be developed with an accuracy equivalent to “ SD BIOLINE HAT.” Such an RDT would have advantages in simplified manufacture, lower unit cost and assured reproducibility.

## Author Summary

The most prevalent species of trypanosome causing human African trypanosomiasis (HAT), *Trypanosoma brucei gambiense*, presents a diagnostic challenge. While early diagnosis is essential for effective treatment and also to control transmission, symptoms are non-specific and parasitological diagnosis is laborious and technically difficult. Screening for HAT suspects has until now been done using the card agglutination test for trypanosomiasis (CATT), which requires a cold chain and equipment, making it difficult to deploy. Thus there is an urgent need for sensitive point of care diagnostic tests that are suitable for use in rural areas in terms of stability, simplicity and cost. We describe the evaluation of 3 rapid diagnostic tests (RDTs) for HAT based on lateral flow devices that detect antibodies against defined parasite antigens in blood samples. We demonstrate that the SD BIOLINE HAT RDT currently being deployed in HAT endemic regions, as well as two new prototype devices, are accurate in screening for HAT. By analysing the sensitivity of each of the antigens used in the devices tested, we predict that a highly sensitive RDT based on recombinant antigens can be developed. An all-recombinant antigen RDT offers significant benefits in manufacturing reproducibility and cost, and would dramatically simplify HAT diagnosis.

## Introduction

Human African trypanosomiasis (HAT), otherwise known as sleeping sickness, is caused by infection with the haemoflagellate parasites *Trypanosoma brucei gambiense* (in west and central Africa) and *T. b. rhodesiense* (in east and southern Africa) [1]. Infection is initiated after the bite of an infected tsetse fly vector and progresses through an “early” stage when parasites proliferate in the haemo-lymphatic system causing a febrile illness, followed by a second or “late” stage of disease in which parasites invade the central nervous system (CNS) causing meningoencephalitis [2]. This latter stage is associated with neurological disturbances and ultimately death [3]. Overall, *T. b. rhodesiense* infections have an acute presentation with the onset of late stage and death within a few months of infection, while *T. b. gambiense* infections are chronic and may persist for several years although there is a spectrum of presentations within each sub-species [4].

Drug treatment, albeit with problems of toxicity, is available for both sub-species [5], and this in combination with control programmes dealing with the vector and infections in zoonotic hosts have reduced the disease prevalence [6]. While the number of reported new cases is now less than 10,000 per year, it is likely that there is considerably greater burden of undiagnosed cases due to diagnostic challenges and inadequate surveillance. The clinical signs of HAT, especially in the early stages, are difficult to distinguish other infectious diseases such as malaria [7]. Initial screening of patients involves indirect diagnostic techniques, the most widely used of which is a serological test, the Card Agglutination Test for Trypanosomiasis (CATT) [7]. This must be followed by parasitological diagnosis, which is laborious, may require concentration techniques due to low parasitaemia, and must be carried out by skilled microscopists.

The CATT is based on the agglutination by serum antibodies of lyophilized bloodstream forms of *T. b. gambiense* expressing variant surface glycoprotein type LiTat1.3, which is expressed widely in *T. b. gambiense* isolates. Using undiluted blood, reported sensitivity varies between 0.688 and 1 and specificity between 0.835 and 0.993 [8]. Cases where specificity and sensitivity are lower are most likely due to exposure of the host to non-pathogenic trypanosomes [9] and infections with clones of *T. b. gambiense* that do not express LiTat1.3 [10], respectively. Although a valuable diagnostic, the CATT does not meet the ASSURED criteria [11] due to a lack of robustness [8], and the production process is also difficult to scale up. Yet, CATT is the only indirect diagnostic test that comes close to meeting the ASSURED criteria. Other immunological and molecular methods that perform well in a laboratory setting are expensive to conduct and require a combination of specialized equipment and skilled personnel (reviewed in [8]). Thus, for the aims of eliminating HAT by 2020 as envisaged by the WHO Roadmap [12] and the London Declaration on NTDs [13] to be achieved, it will be essential to develop ASSURED compliant tests that are easy to produce at scale.

Immunochromatographic lateral flow devices are capable of detecting low concentrations of antibodies to target antigens in biological fluids [14], [15]. This technology may be used to develop rapid diagnostic tests (RDTs) that can detect anti-trypanosome antibodies in finger prick samples of human blood. RDTs based on lateral flow devices are simple to use, easy to read and have stability characteristics that allow distribution and availability in remote endemic areas. Recently the first RDT for HAT was deployed in the field. The test, developed by the Foundation for Innovative New Diagnostics (FIND) and Standard Diagnostics (SD BIOLINE HAT), is based on a device using native variant surface glycoproteins (VSG) LiTat1.3 and LiTat1.5 to detect antibodies to trypanosomes [16]. A further lateral flow RDT based on these antigens (HAT Sero-*K*-SeT) has been described and developed by Coris Bioconcept [17].

In this paper we describe two further devices developed by SD and BBI Solutions (UK). The first uses recombinant LiTat1.3 and LiTat1.5 antigens. While these are the same antigenic targets as used in the SD BIOLINE HAT, HAT Sero-*K*-SeT and CATT (LiTat1.3) tests, the use of recombinant antigens has potential to simplify the production and reduce the costs of RDTs. The second prototype device, that uses the diagnostic potential of ISG65 [18], is based on a combination of recombinant ISG65 and a native VSG MiTat1.4 [19]. ISG65 is one of two well-characterised moderately abundant invariant type-1 trans-membrane domain surface glycoproteins that is expressed in *Trypanosoma brucei* [20]. A summary of the three RDTs studied here is presented in Table 1.

**Table 1:** Antigens used in the three RDTs.

RDT	Band 1	Band 2	Reference
NatSD	LiTat1.3	LiTat1.5	[16]
RecSD	rLiTat1.3	rLiTat1.5	Not published
BBI	rISG65	MiTat1.4	[19]

The aim of this study was to evaluate the performance of the two new prototype RDTs in comparison to SD BIOLINE HAT in a side-by-side analysis using archived plasma samples from HAT patients and endemic controls.

## Methods

### Study Design

This was a retrospective study. Clinical samples of heparinised plasma were obtained from 250 *T. b. gambiense* patients and 250 endemic controls. The sample size of infected and control groups was calculated to detect a 5% performance margin between devices at a power of 0.8 and confidence level of 0.95. The samples were obtained from FIND-sponsored field studies in Angola, Central African Republic (CAR) and Uganda, and held in cryobanks in Makerere University (Uganda) and the University of Limoges (France). Demographic details of the patient and control cohorts are presented in S1 Table. The infection status of patients was confirmed by observation of parasites in the blood, lymphatic system or cerebrospinal fluid, and this provided the reference standard. Patient samples were collected consecutively and there was no further selection for the purpose of this study. Controls were CATT negative and had no history of HAT or evidence of trypanosomes in blood when tested using the miniature anion exchange centrifugation test (mAECT). After collection, samples from Angola and Uganda were kept in liquid N_2_ in the field and during transportation, and then stored frozen at −80°C. In CAR samples were kept at +4°C in the field and transferred to a central laboratory within 14 days where they were stored at −80°C. Samples were sent frozen on dry ice to the University of Dundee where they were blinded and randomised, and then to the University of Aberdeen for testing with the RDTs. The readers in Aberdeen were blind to the status of all samples.

### Ethics Statement

All clinical samples were obtained after written informed consent. Country-specific study protocols were approved by the following institutional review boards: Comissão de Ética do Instituto de Combate e Controlo das Tripanossomiases (Angola, Meetings 12/02/08 and 12/07/11), Comité scientifique chargé de la validation des proto-coles d'études et des résultats de la Faculté des sciences de la santé de l'Université de Bangui (CAR, 9/UB/FACSS/CSCVPER/12) and Uganda National Council for Science and Technology (HS 792).

### Rapid Diagnostic Tests

Three RDTs were used in this study (\).

- A registered and commercialized RDT (“SD BIOLINE HAT”) manufactured by Standard Diagnostics, Inc. (SD) that is based on two native VSGs LiTat 1.3 and VSG LiTat 1.5 antigens (hereafter NatSD)
- A prototype RDT developed by SD that is based on Baculovirus-expressed recombinant VSG LiTat 1.3 and VSG LiTat 1.5 antigens (RecSD)
- A prototype RDT developed by BBI Solutions that is based on recombinant ISG65 and native VSG MITat 1.4 antigens (BBI).

**Figure 1:**
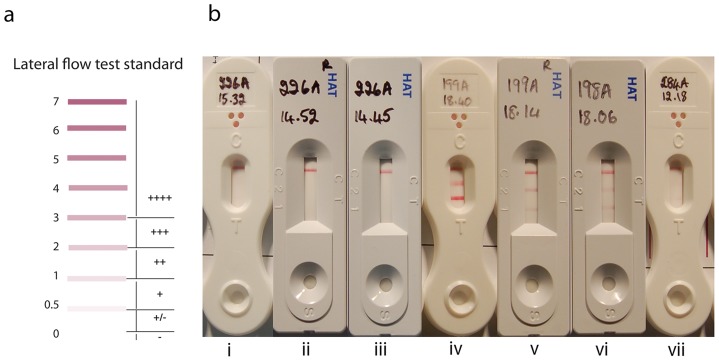
(a) 4-point-standard reference card for scoring RDT bands. Bands were assessed in realtion to the -, +, ++, +++ or ++++ ranges on the reference card, and then converted to scores of 0, 1+, 2+, 3+ and 4+ respectively. Additionally faint bands (+/-) were recorded as 0.5 and unless otherwise stated scored as sub-threshold (0). (b) Examples of positive and negative results with each device. Negative devices are presented in panels (i)–(iii): (i) BBI, (ii) RecSD (iii) NatSD. In all of these devices the bands are scored: 4+, 0, 0 (control, band 2, Band 1). Positive devices are presented in panels (iv)–(vi.) (iv) BBI bands were scored: control  =  4+, 3+, 4+ (v) RecSD bands were scored: 4+, 3+, 1+ (vi) NatSD bands were scored: 4+, 2+ 2+. (vii): Example of a BBI device scored as negative (4+, 0, 0) but where the readers noted faint bands (+/-) in positions 1 and 2.

Each device was run according to the manufacturers recommendations. Freshly thawed plasma (10 µl for SD devices, 5 µl for BBI devices) was applied to the sample well, followed by chase buffer (120 µl for SD devices, 95 µl for BBI devices). Each plasma sample was applied to duplicate devices. The devices were incubated for 15 min (NatSD and RecSD) or 30 min (BBI) at room temperature. After the incubation period, each device was read by visual comparison using a 4-point lateral flow test standard (Fig. 1). The appearance of antigen bands (bands 1 and 2) was scored as 0, 1+, 2+, 3+ or 4+ depending on colour intensity. Additionally, where readers detected a faint band that was judged below the threshold of the 4-point standard (+/-), the result was annotated with a score of 0.5 and used in a reanalysis of the accuracy of each device. The 3^rd^ band on each device was a control band. Devices (4/1000 BBI devices and 1/1000 RecSD device) where no control band was observed were discarded and the test repeated on a new device.

Each RDT was scored independently by each of two readers. The readers were not aware of each other's scores until they had both been recorded. Primary and secondary readings took place within 5 minutes of each other.

### Data Analysis

After all plasma samples had been run and scored, the raw data were sent to Dundee University for the sample codes to be un-blinded and identified as infected or control. Each score (0, 1+, 2+, 3+ 4+) was represented by an integer between 0 and 4. In a second run analysis we rescored all the bands that had been annotated as faint and below threshold as 1. We established an arbitrary limit, *L*, to decide whether a score is positive or negative. A score was considered positive if it was greater than or equal to *L*. Unless otherwise stated in the results, sensitivity, specificity and accuracy were calculated at the cut-off level of *L*  = 1. When two antigen bands were read from a single device, the result was considered positive if either of the scores was positive. These positives and negatives were then compared with patient data and the total counts of true positives (TP), false positives (FP), true negatives (TN) and false negatives (FN) were summed across all patients for each reader and each duplicate device. The sensitivity, specificity and accuracy were defined as
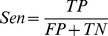


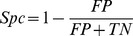


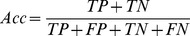



respectively. These can be alternatively defined as true and false positive rates, where *TPR*  =  *Sen* and *FPR*  = 1− *Spc*, respectively. For the given reader and duplicate device, the errors on the above quantities were found as 95% confidence intervals of a proportion [21]. When reader data were combined, the counts of *TP*, *FP*, *TN* and *FN* were averaged across both readers and duplicate devices, which were considered as a set of 4 replicates. The mean and its 95% confidence interval were found across these replicates and errors were then propagated to *Sen*, *Spc* and *Acc*.

The diagnostic results from each plasma sample using replicate devices or between reader 1 and 2 were tested for agreement using Cohen's kappa (*k*). To compare the duplicate devices, we aggregated data from both readers and vice versa, to compare the readers, we aggregated data from both duplicate devices. Uncertainties of *k* were estimated following Fleiss *et al*. [22].

All errors quoted in this work are 95% confidence intervals. The difference between the means is assessed by a *t*-test (assuming equal variance) at a significance level of 0.05.

## Results

### Inter-Reader and Inter-Device Agreement

Examples of each RDT used in this study, in which the different scores (faint-sub-threshold, 1+, 2+, 3+, 4+) and the difference between typical positive and negative results, are illustrated in Fig. 1.

**Figure 2:**
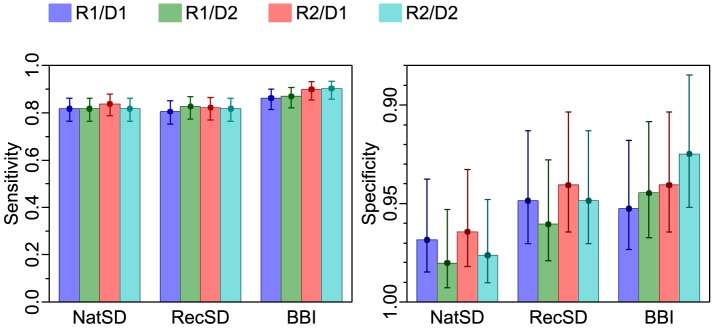
Sensitivity and specificity of devices by each reader (R1 and R2) and by duplicate device (D1 and D2). Error bars are 95% confidence intervals of a proportion.

Following scoring of the randomised and blinded groups of 250 HAT patient plasmas and 250 endemic control plasma samples, the sensitivity and specificity of each RDT was calculated for each reader and each duplicate test. The results presented in Fig. 2 demonstrate close agreement between readers and duplicate assays. The level of agreement was further quantified using Cohen's *k* (Table 2). A value of *k* ≥0.9 was found for all inter-duplicate and inter-reader agreements, which represents a very good level of agreement [23]. As there were no significant differences between the diagnostic results of the two readers using the minimum visual score of 1+ for a positive, the duplicate readings by each reader were re-analysed as four replicates and formed the basis of further analysis of RDT and individual antigen performance.

**Table 2:** Cohen's kappa and its 95% confidence intervals calculated for each RDT between duplicate devices and between independent readers.

RDT	Inter duplicate RDT	Inter reader
NatSD	0.96±0.02	0.97±0.02
RecSD	0.94±0.02	0.96±0.02
BBI	0.91±0.03	0.93±0.02

### Performance of Each RDT

**Figure 3:**
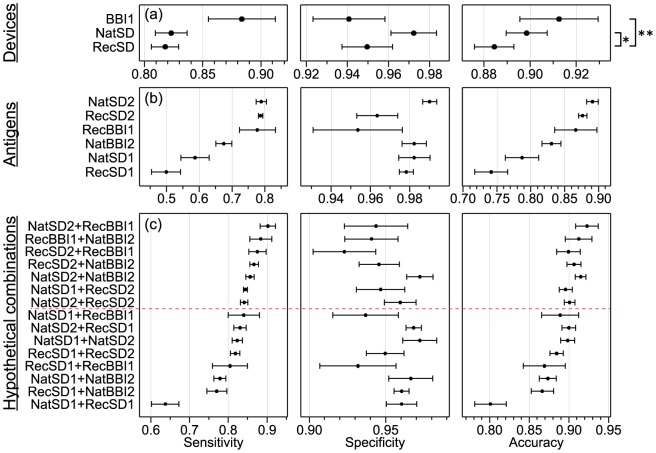
Performance of (a) each RDT, (b) each antigen, and (c) all pairwise combinations of antigens, ordered by sensitivity. Error bars represent 95% confidence intervals, derived from 4 replicates (2 readers using 2 duplicate RDTs). The asterisks in (a) indicate statistically significant difference between the mean accuracy (from a t-test) of p* = 0.01 and p** = 0.003. The antigen combinations above the dashed horizontal line in (c) have significantly better sensitivity to NatSD device.

The sensitivity, specificity and accuracy of each device are presented in Fig. 3a and Table 3. Both prototype devices are not inferior to the NatSD RDT in any of these three parameters at the required 5% margin. The sensitivity of the BBI device (0.88±0.03) is however significantly superior to both NatSD and RecSD (both 0.82±0.01), with *p* = 9×10^−4^ and 5×10^−4^, respectively. All devices show a performance similar to or better than the range of sensitivity (≥0.7) and specificity (≥0.8) reported for the CATT [8]. The specificity of the NatSD RDT is highest (0.97±0.01) but not significantly superior to the other devices. The accuracy of both the prototype BBI (0.91±0.02) and NatSD (0.898±0.009) devices are significantly higher than the accuracy of the RecSD device (0.884±0.008), with *p* = 0.003 and 0.01, respectively, and we found no evidence for the BBI test having different accuracy from the NatSD RDT (*p* = 0.06).

**Table 3:** Sensitivity, specificity and accuracy of RDTs.

RDT	Sensitivity	Specificity	Accuracy
NatSD	0.82±0.01	0.97±0.01	0.898±0.009
RecSD	0.82±0.01	0.95±0.01	0.884±0.008
BBI	0.88±0.03	0.94±0.02	0.91±0.02
CATT^a^	≥0.7	≥0.8	

Errors are 95% confidence intervals.

a For CATT an approximate range of reported sensitivity and specificity is quoted after [8].

### Effect of Scoring Any Faint Band as Positive (1+)

When scoring the RDTs in this trial, each reader also made a record of any faint bands in the sub-threshold range (+/-) on the reference card (Fig. 1a). These were given a nominal score of 0.5, and were therefore below the cut off limit (*L* = 1) for a positive result. In order to determine the effect of including such faint bands as positive they were rescored as 1+. When this was done there was an increase in sensitivity for all devices with a loss of specificity (Table 4). This was most pronounced with the BBI device with a sensitivity of 0.96±0.03 (an increase of 8%) but a specificity of 0.79±0.15 (a loss of 15%). While the recording of sub-threshold bands marginally increased inter-device agreement for duplicate devices, it led to a considerable reduction of inter-reader agreement (Table 5) especially in the case of the BBI device.

**Table 4:** Sensitivity, specificity and accuracy of RDTs after sub-threshold faint bands (+/-) were scored as positive (1+).

RDT	Sensitivity	Specificity	Accuracy
NatSD	0.89±0.03	0.91±0.06	0.90±0.03
RecSD	0.91±0.05	0.88±0.07	0.90±0.04
BBI	0.96±0.03	0.79±0.15	0.87±0.08

Errors are 95% confidence intervals.

**Table 5:** Cohen's kappa and its 95% confidence intervals calculated for each RDT between duplicate devices and between independent readers after sub-threshold faint bands (+/-) were scored as positive (1+).

RDT	Inter duplicate RDT	Inter reader
NatSD	0.97±0.02	0.88±0.03
RecSD	0.97±0.01	0.86±0.03
BBI	0.93±0.02	0.76±0.04

### Individual Antigen Performance

The scores recorded for each band on the 3 devices allowed the diagnostic potential of each of the 6 antigens to be evaluated (Fig. 3). This analysis reveals that NatSD2 (LiTat1.5) provides the best diagnostic performance, with the highest sensitivity, specificity and accuracy. This is followed by RecSD2 (rLiTat1.5) and RecBBI1 (rISG65), which had a comparable sensitivity, but a poorer specificity.

### Predicted Performance of All Antigen Combinations

The performance of each of the antigens was used to predict the theoretical performance of all combinations of 2 antigens on hypothetical new RDT formulations (Fig. 3c). This analysis provides evidence that new antigen pairs have the potential for use in developing new improved RDTs. On examination, 6 novel combinations and the BBI device out-perform the NatSD RDT, providing significantly better sensitivity (data above the dashed line in Fig. 3c). The top combination with the highest sensitivity and accuracy is NatSD2+RecBBI1 (LiTat1.5+rISG65), though neither its sensitivity (0.90±0.02) nor accuracy (0.92±0.01) is superior to the prototype BBI device (*p*>0.1 in both cases). A combination of NatSD2+NatBBI2 (LiTat1.5+MiTat1.4) is among those with the highest specificity (0.972±0.009) while retaining a high sensitivity (0.86±0.01). The optimal pairing of recombinant antigens is RecSD2+RecBBI1 (rLiTaT1.5+rISG65), whose accuracy of 0.90±0.02 is not significantly different to the BBI device evaluated here.

A similar analysis of performance of hypothetical 3 antigen band multiplex lateral flow devices for all three-way combinations of antigens was carried out and demonstrated no significant improvement in performance over the 2 antigen devices (S1 Fig.).

### Partial ROC Curves

The scores provided by readers for each antigen band are not binary, but take into account the intensity of the band, comprising a scale between 0 and 4. In the analysis so far, we converted them into positives and negatives using a fixed limit of *L* = 1. In other words any band scored by matching the colour scale (Fig. 1a) as 1+ or greater is scored as positive. By increasing this limit we can study the effects of deteriorating field conditions such as poor lighting or reader eyesight in which weak bands may not be recognized.

**Figure 4:**
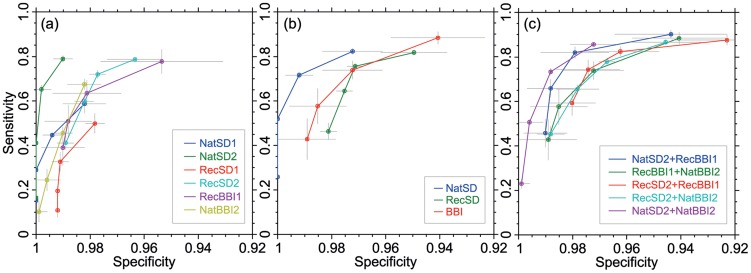
Partial receiver operating characteristic (ROC) curves for (a) individual antigens, (b) RDT devices and (c) top six performing hypothetical combinations of two antigens. Each curve represents a changing antigen band intensity limit from *L* = 1 (top right) to *L* = 4 (bottom left). Error bars are 95% confidence intervals.

Fig. 4 shows the effect of varying *L* on sensitivity and specificity. The partial receiver operating characteristic (ROC) curves were calculated for the cut-off from *L* = 1 (top right) up to *L* = 4 (bottom left). We note that due to a very limited range of specificity, we cannot reliably calculate the area under the curve. With increasing *L* (corresponding to deteriorating field conditions) there is an often dramatic drop in sensitivity, as fainter antigen bands are not spotted. On the other hand, there is a corresponding increase in specificity, as the faintest bands can create false positives.

Of the individual antigens (Fig. 4a), RecSD2 (rLiTat1.5) and RecBBI1 (rISG65) show the most moderate loss of sensitivity, down to ∼0.4. The other four antigens drop in sensitivity below 0.2 in the limit of *L* = 4. Fig. 4b shows that the NatSD RDT, while displaying consistently highest specificity, loses more sensitivity with deteriorating conditions than RecSD and BBI. Fig. 4c shows a selection of six hypothetical antigen combinations with highest sensitivity (cf. Fig. 3c). The combination of the two antigens with the lowest loss of sensitivity is RecSD2+RecBBI1 (rLiTat1.5 and rISG65).

## Discussion

This study aimed to evaluate the performance of two novel prototypes and the commercially available SD BIOLINE HAT (NatSD) RDT in a side-by-side analysis using a panel of archived plasma samples from HAT patients and endemic controls. The sample size was designed using power analysis to be able to detect an inferiority margin of 5%. Evaluation of the RDTs was carried out by two readers in a blinded manner at a separate institution remote from where the un-blinded sample identities were held, and the two readers scored each device entirely independently of each other's readings. The samples were classified in the field at the time of collection as infected or control on the basis of robust criteria. For infected individuals, while initial identification of suspects was via the CATT test and presenting symptoms, all cases were confirmed parasitologically. All the controls had no symptoms, were negative with CATT, and had no detectable trypanosomes in the blood after the use of concentration techniques. It is possible that within this group there could have been sub-clinical cases with a very low parasitaemia, particularly if they were from parasites that did not express the CATT antigen (LiTat1.3) or from individuals who were immunologically unresponsive to that antigen. We consider this unlikely, and indeed it may be predicted if that was the case then the RDT bands using a non-variant antigen (BBI1/ISG65) and a non-CATT antigen (LiTat1.5, NatSD2, RecSD2) would exhibit a higher specificity. The data (Table 3) did not support this prediction.

With all 3 RDTs a very high level of agreement (Cohen's *κ*≥0.9) was obtained between readers and also between the duplicate RDTs used with each sample. Inter-reader agreement is in fact better than for CATT (*κ* = 0.84, [24]) and also for a recent laboratory trial implementation of the loop-mediated isothermal amplification (LAMP) diagnostic [25].

On the basis of these results, duplicate readings by each reader were treated as replicates for the performance evaluation of each device.

The sensitivity, specificity and accuracy were calculated for each device. All the devices performed well, and while the prototypes were not inferior at the 5% level in terms of sensitivity, specificity and accuracy in comparison with the NatSD, the BBI prototype was significantly more sensitive than the NatSD RDT. The sensitivity and specificity compared well to the range of published performance of the CATT (sensitivity 0.69–1.0, specificity 0.84–0.99 [8]) and LAMP (sensitivity 0.87–0.93, specificity 0.93–0.96 [26]). When overall accuracy was calculated, there was no significant difference in performance between the NatSD RDT and the BBI prototype, but the RecSD prototype was significantly inferior to both.

While sensitivity in this blinded study of both NatSD RDT and the BBI prototype were 0.82±0.01 and 0.88±0.03 respectively, a field trial study of the Coris Bioconcept HAT Sero-*K*-SeT lateral flow device [17] has recently been reported to give a sensitivity of 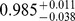 and a specificity of 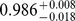 (95% CI) [27]. While different lateral flow platforms are used in NatSD and HAT Sero-*K*-SeT, they use the same antigens for detection. There are two possible reasons for the apparent discrepancy between the results for the RDTs presented here and those obtained with the HAT Sero-*K*-SeT. First, in this study archived plasma was used rather than whole blood. First, in this study archived plasma was used rather than whole blood. While there have been no published systematic side-by-side studies of the impact of this difference in immunodiagnostic assays for HAT, it is possible that performance of the tests would be improved when fresh blood samples are used. Secondly, there were important differences in the observation methodology. While in this study, all the results were scored completely blind, for fully described clinical and operational reasons [27] in the evaluation of HAT Sero-*K*-SeT about half of the samples were scored by readers already knowing a parasitological diagnosis or being aware of the clinical signs of the subjects. This has the potential to bias decisions on the reading of faint bands according to the known diagnosis or symptomatology of the subject, thus increasing the apparent sensitivity and specificity of the test. To model the effect of including all faint bands with the devices in this study, we reanalysed our data scoring every band that had been annotated sub-threshold as 1+. This led to a significant increase in sensitivity with a performance of the BBI RDT that was not statistically different to the HAT Sero-*K*-SeT. In this case there was naturally a loss of specificity, as all faint bands were scored as positive. The scoring of faint bands led to a reduction of inter-reader agreement, and this is likely to be due to differences in visual acuity of different readers, given that both readers worked under identical lighting conditions. This reduction of inter-reader agreement justifies the use of the cut off of 0.5 (*L* = 1) on the 4 point reference card, as sensitivity data obtained by scoring very faint bands as positive would not be reliably be duplicated by other readers.

The current diagnostic procedure for HAT includes identification of suspects using a screening test, followed by parasitological confirmation [7]. This is essential, first to ensure that subjects who are false positive with the screening test do not undergo uncomfortable lumbar puncture during staging, and secondly to avoid exposing them to drug treatments that are associated with toxicity [5]. Therefore in assessing the performance of RDTs, the most important criterion is high sensitivity, as the false positives resulting from lower specificity may be excluded during parasitological confirmation. In this respect, the BBI prototype out-performed the others used in this study. It exhibited a higher sensitivity than both the RecSD and NatSD prototypes, despite a small loss of specificity (less than 5% inferiority margin), and thus would be best placed to take forward for further development. This device has a further advantage over the other devices through its use of a non-variant antigen (ISG65) that would be expressed in all isolates of *T.b. gambiense*, thus theoretically allowing higher sensitivity across a range of diverse *T.b. gambiense* foci. In comparison LiTat1.5 and LiTat1.3, despite having been demonstrated to be very widely expressed [28], will probably not be universally found in variant antigen repertoires as has been demonstrated in the field [10].

When the performance of individual antigens was analysed, the best antigen was NatSD2 (LiTat1.5), followed by RecSD2 (rLiTat1.5) and then RecBBI1 (rISG65). Thus, at the individual antigen level, both the native and recombinant forms of LiTat1.5 were good diagnostic antigens. By increasing the cut-off limit at which an antigen band was considered positive, we demonstrated a deterioration of the performance of the antigens. This reflects the situation that could be encountered in the field if those performing the test are either not adequately trained, or they have other challenges. For example, if the results were read by a person with poor eyesight or the lighting is poor, the weakest antigen band may not be spotted, that otherwise would have been scored as 1. In relation to this, we found that RecSD2 (rLiTat1.5) and RecBBI1 (rISG65) lose much less of their sensitivity than the other antigens.

Based on the performance of the individual antigens, it was possible to predict the performance of all 2-way and 3-way combinations in hypothetical novel multiplex-RDTs [15], based on the assumption that the antigens behave identically in performance in different combinations. This is a powerful approach to selection of antigens that should be exploited in development of the next generation of RDTs for HAT. Of the hypothetical devices, none of the 3-antigen combinations were superior to 2-antigen devices. Of the 2-antigen devices tested here, this analysis suggests that the combination in the BBI prototype (LiTat1.5 and rISG65) is the best. However this device includes a native antigen, which presents some production and manufacturing difficulties. Yet, when we examined the hypothetical performance of devices with recombinant antigens only, it was apparent that one with rLiTat1.5 and rISG65 (RecSD2+RecBBI1) would have a performance similar to the current best RDTs and would have an advantage of the smallest drop in sensitivity under deteriorating field conditions. Because recombinant antigens offer significant advantages in device manufacturing and reproducibility, we suggest these two antigens as important candidates for consideration in development of the next generation of RDTs for HAT.

## Supporting Information

S1 Fig Performance of all 3-way combinations of antigens, ordered by sensitivity. Error bars represent 95% confidence intervals, derived from raw performance data of individual antigen band comprising 4 replicates (2 readers using 2 duplicate RDTs).
(EPS)

S1 Table **Details of samples.**
(DOCX)

S1 Checklist **STARD checklist.**
(DOC)
