# Correction: Evaluation of the Diagnostic Accuracy of Prototype Rapid Tests for Human African Trypanosomiasis

**DOI:** 10.1371/journal.pntd.0003613

**Published:** 2015-03-17

**Authors:** The PLOS Neglected Tropical Diseases Staff

There are two errors in this article.

In the first sentence of the Methods section under the heading “Rapid Diagnostic Tests”, the text in the parentheses is incorrect. The correct text should be Table 1.

In the Data Analysis section, there is an error in the first equation. The quantity in the denominator is incorrect. It should read TP+FN. Please view the complete, correct equation here:

$$Sen=\frac{TP}{TP+FN}$$
