# Supplementary material for: Evaluation of the Diagnostic Accuracy of Prototype Rapid Tests for Human African Trypanosomiasis
Source: PLoS Negl Trop Dis. 2014 Dec 18;8(12):e3373. doi: 10.1371/journal.pntd.0003373 (PMC4270746; doi:10.1371/journal.pntd.0003373)
Supplement: S1 Table — Details of samples. (DOCX) [file pntd.0003373.s002.docx]

**Supplementary Table 1**

Details of samples

| Country and site of clinic | Number of infected | Age range of infected | Gender Ratio of infected (male/female) | Presenting symptom spectrum | Number of controls | Age range controls | Gender ratio controls |
| --- | --- | --- | --- | --- | --- | --- | --- |
| Uganda(Omugu Heatlh Centre) | 61 | 1-55 | 1.18 | Not available | 70 | 3-58 | 0.75 |
| CAR | 73 | 1-75 | 0.74 | Arthralgia, myalgia, weight loss, asthenia, fever, impotency, headache, sleeping disorders, pruritus, walking disorders, loss of appetite, convulsions, speech disorders | 0 | - | - |
| Angola | 126 | 10-66 | 0.75 | Sleep disturbance, behaviour disturbances, cognitive disturbance, depression, hypersensitivity, Romberg’s sign, hyperesthenia, headache, abnormal movements, tremor, extrapyramidal syndrom | 190 | 10-87 | 1.1 |
